# Attenuation of Tau-Mediated β-Amyloid1-42 Aggregation by Native PLGA Nanoparticles and Its Relevance to Alzheimer Disease Pathology

**DOI:** 10.3390/ijms27188152

**Published:** 2026-09-13

**Authors:** Pallabi Sil Paul, Aliza Borenstein-Katz, Klinton Shmeit, Holger Wille, Satyabrata Kar

**Affiliations:** 1 Department of Medicine (Neurology), Centre for Prions and Protein Folding Diseases, University of Alberta, Edmonton, AB T6G 2M8, Canada; pallabis@ualberta.ca; 2 Department of Biochemistry, Centre for Prions and Protein Folding Diseases, University of Alberta, Edmonton, AB T6G 2M8, Canada; akatz@ualberta.ca (A.B.-K.); shmeit@ualberta.ca (K.S.); wille@ualberta.ca (H.W.); 3 Department of Psychiatry, Centre for Prions and Protein Folding Diseases, University of Alberta, Edmonton, AB T6G 2M8, Canada

**Keywords:** Alzheimer’s disease, tau seeds, tau monomer, PLGA nanoparticles, Aβ aggregation

## Abstract

Alzheimer’s disease (AD) is an unremitting neurodegenerative disorder characterized by the presence of extracellular β-amyloid (Aβ)-containing neuritic plaques, intracellular tau-positive neurofibrillary tangles and loss of selected neurons in the brain. Evidence suggests that aggregation of Aβ and tau via synergistic interactions initiates a cascade of events, leading to development of AD pathology. Thus, many studies are being pursued to develop small molecules/drugs that can target both Aβ and tau as an effective AD treatment strategy. In this study, we evaluated effects of native PLGA nanoparticles on tau-mediated Aβ1-42 aggregation using biophysical, structural, spectroscopic and biochemical approaches. Our results show that 0N4R tau seeds enhanced Aβ1-42 aggregation, and that this effect is mitigated by native PLGA. Additionally, PLGA inhibited Aβ1-42 aggregation induced by both 0N4R and 2N4R tau isoforms. The presence of PLGA during the formation of tau seeds or pretreatment of tau seeds with PLGA attenuates subsequent Aβ1-42 aggregation. Interestingly, monomeric 0N4R tau, unlike 0N4R tau seeds, suppressed Aβ1-42 aggregation, which is diminished further by PLGA nanoparticles. However, we have not addressed the implications of native PLGA on tau/Aβ1-42 interaction using any cellular or animal models of AD. Nevertheless, our results reveal that tau seeds and monomeric tau can differentially influence Aβ1-42 aggregation, which is mitigated by native PLGA under in vitro conditions, providing a rationale to study it further under an in vivo paradigm to examine its significance in AD pathogenesis.

## 1. Introduction

Alzheimer’s disease (AD) is an unremitting neurodegenerative disorder characterized pathologically by the presence of extracellular neuritic plaques, intracellular neurofibrillary tangles (NFTs) and the loss of neurons in defined regions of the brain [1,2,3]. While neuritic plaques primarily comprise β-amyloid (Aβ) peptides generated from amyloid precursor protein (APP), the NFTs are formed by the hyperphosphorylation of microtubule-associated tau protein. Evidence suggests that proteolytic processing of APP via β- and γ-secretases can generate various Aβ peptides containing 39–43 amino acids, but the two most prevalent isoforms in the normal brain are Aβ1-40 and Aβ1-42 [4]. Of the two major isoforms, Aβ1-42 constitutes ~10% of the total Aβ secreted from cells but aggregates faster and is more toxic to neurons than Aβ1-40. A progressive increase in brain Aβ1-42 levels, resulting from enhanced production and/or decreased clearance, is believed to trigger the conversion of soluble Aβ1-42 monomers into aggregated forms, leading to the development of AD pathology [5,6]. This is supported by data showing that accumulation of intracellular Aβ occurs prior to any other pathology, and soluble oligomers/aggregates can cause neuronal dysfunction leading to death in AD brains [7]. Thus, preventing Aβ aggregation into toxic fragments is considered to be a promising strategy for preventing or delaying the onset of AD.

Notwithstanding the critical role of Aβ1-42 in disease pathology, the number of tau-positive NFTs correlates with the clinical severity of dementia and the loss of neurons associated with the AD brain [3,4]. Tau is a soluble protein, evident mostly in neurons, comprising an N-terminal domain, proline-rich domain, microtubule-binding domain (MTBD) and C-terminal domain. It exists in six different isoforms ranging from 352 to 441 amino acids containing 0, 1, or 2 N-terminal repeats (0N/1N/2N) with 3- or 4-MTBD repeats (4R/3R) [8]. Under physiological conditions, tau plays an important role in stabilizing microtubules, regulating intracellular trafficking via a site-specific phosphorylation-dephosphorylation process mediated by multiple kinases and phosphatases, respectively. Enduring hyperphosphorylation, however, promotes detachment of tau from microtubules, leading to its fibrillization into paired helical filaments (PHFs) and deposition as NFTs in AD brains [9,10,11,12]. There is evidence that Aβ peptide can induce phosphorylation and aggregation of tau protein, which can lead to degeneration of neurons, suggesting synergistic interactions rather than distinct effects of either Aβ or tau protein may underlie the development/progression of AD pathology [1,2,3,4,13]. This is supported by data showing that (i) Aβ toxicity is not mediated in the absence of tau protein, (ii) Aβ aggregates can trigger/propagate tau pathology in cultured cells and animal models, (iii) Aβ-derived peptidomimetics can inhibit both Aβ and tau aggregation, (iv) tau-derived peptidomimetics and native tau can differentially alter Aβ aggregation, (v) the Aβ core region can bind directly to tau and (vi) complexes of Aβ bound to tau are detected in AD brain extracts [14,15,16,17,18,19,20]. Given the entwined nature of these two proteins, it is essential not only to understand better how their functions are integrated, but also to develop a treatment strategy that can target both proteins simultaneously in AD brains.

Over the years, several molecules/agents, including phytochemicals (resveratrol, curcumin, and quercetin), hormones (estradiol, and melatonin), metal chelators, and antibodies, have been explored as novel therapeutics to arrest the progression of AD. These agents are transported across the blood–brain barrier using various nanoparticles, which are engineered materials >100 nm in diameter with unique physiochemical properties, to attenuate disease pathology by regulating Aβ or tau aggregation and toxicity [21,22,23,24,25]. Among numerous families of nanoparticles, FDA-approved acidic poly (D,L-lactide-co-glycolide) (PLGA) nanoparticles, synthesized from glycolic acid and lactic acid, have become ubiquitous as a delivery vehicle for various drugs/agents because of their biocompatibility and excellent safety profile. In fact, PLGA-conjugated drugs/agents such as donepezil, memantine, curcumin, galantamine, quercetin and selegiline have been shown to exhibit beneficial effects on Aβ or tau pathology in a variety of cellular/animal models of AD [26,27,28,29,30,31,32]. Notwithstanding these results, we reported that PLGA nanoparticles without conjugation with any drug/agent (i.e., native PLGA) can also attenuate Aβ1-42 aggregation/toxicity under in vitro conditions [33,34,35]. More recently, we have shown that native PLGA can suppress Aβ1-42 seed-induced tau aggregation [36]. However, it remains unclear whether Aβ1-42 aggregation can be influenced by a distinct isoform/conformation of tau that can initiate a feedback cascade prompting the development/progression of AD pathology. In this study, we showed that 0N4R and 2N4R tau seeds, in contrast to monomeric tau, can markedly enhance Aβ1-42 aggregation, which can be decreased by native PLGA, highlighting not only the synergistic interactions between Aβ1-42 and tau, but also the implication of native PLGA in the treatment of AD.

## 2. Results

### 2.1. Effect of 0N4R Tau-Seed on Aβ1-42 Aggregation

**Figure 1:**
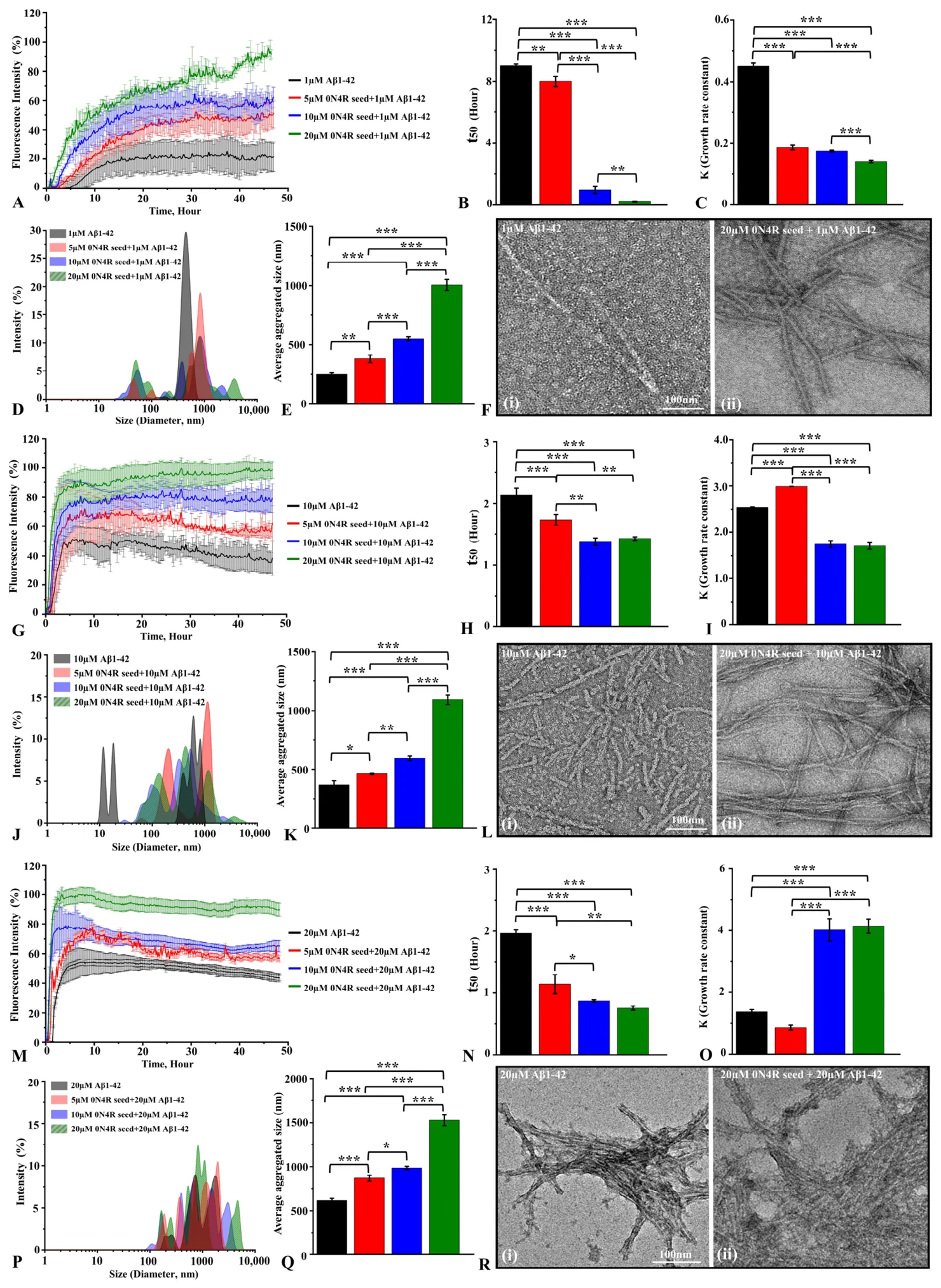
0N4R tau-seed-induced Aβ1-42 aggregation. (**A**–**R**): ThT kinetic assays showing aggregation curves (**A**–**C**,**G**–**I**,**M**–**O**), and the corresponding DLS analysis (**D**,**E**,**J**,**K**,**P**,**Q**) as well as TEM (**F**,**L**,**R**) images of 1 µM Aβ1-42 (**A**–**F**), 10 µM Aβ1-42 (**G**–**L**) and 20 µM Aβ1-42 (**M**–**R**) in the presence or absence of 5, 10 and 20 µM 0N4R tau seeds over 48 h incubation at 37 °C. Note the aggregation of Aβ1-42 increases as a function of tau seed concentrations, which reaches plateaus over time as indicated by ThT fluorescence levels. Fitting of the kinetic curves using a logistic IV function showed a progressive decrease in t_50_ for all Aβ1-42 concentrations with increasing tau seed levels (**B**,**H**,**N**). The apparent growth rate constant k decreased for 1 and 10 µM Aβ1-42 (**C**,**I**), but increased for 20 µM Aβ1-42 (**O**) with enhanced concentrations of tau seeds. DLS analysis demonstrated an overall increase in the hydrodynamic diameter of aggregated Aβ1-42 as a function of tau seed concentrations as shown by size distribution plots (**D**,**J**,**P**) and the corresponding histograms (**E**,**K**,**Q**). Representative TEM images (**F**,**L**,**R**) depicting Aβ1-42 aggregation in the absence and presence of increasing concentrations of tau seeds following 48 h incubation. Note the increased aggregation of Aβ1-42 in the presence of tau seeds (**Fii**,**Lii**,**Rii**) compared to those observed in the absence of tau seeds (**Fi**,**Li**,**Ri**). All ThT kinetic graphs represent mean ± SEM of three separate experiments, each performed with six replicates for each condition. n.s. represents non-significant. * *p* < 0.05, ** *p* < 0.01, *** *p* < 0.001.

A number of earlier studies have shown that Aβ seeds can induce tau aggregation [15,16,36] but the effects of tau seeds on Aβ aggregation remain unclear. Thus, we first performed thioflavin T (ThT) kinetics assays by incubating 1–20 µM Aβ1-42 in the presence or absence of freshly prepared 5, 10, and 20 µM 0N4R tau seeds at 37 °C for 48 h (Figure 1A–R; Supplementary Figures S1A–C and S2A–C). In all experimental conditions, Aβ aggregation displayed a characteristic sigmoidal profile, comprising a lag phase, rapid growth (log) phase, and saturation phase. The presence of tau seeds significantly enhanced the aggregation kinetics of Aβ1-42 in a concentration-dependent manner, as reflected by the shorter lag times and higher plateau of ThT fluorescence compared to Aβ alone (Figure 1A,G,M). Normalized ThT kinetic curves were fitted with a logistic function to determine the half-time (t_50_) and the apparent growth rate constants (k) of 1, 10 and 20 µM Aβ1-42 aggregation in the presence or absence of increasing (5, 10 and 20 µM) 0N4R tau seed concentrations (Figure 1B,C,H,I,N,O, Supplementary Figure S2A–C). Across all three Aβ1-42 concentrations, increasing amounts of tau seeds progressively decreased t_50_, indicating that tau fibrils efficiently accelerate Aβ aggregation kinetics by circumventing the slow primary nucleation step. At 10 µM Aβ1-42, t_50_ decreased from ~2.1 h (Aβ alone) to ~1.7–1.4 h in the presence of 5–20 µM tau seeds. A similar monotonic reduction was evident at 1 µM and 20 µM Aβ1-42, where t_50_ decreased from ~9 and ~2 h, respectively, to below ~1 h at the highest seed concentrations (Figure 1B,H,N). The apparent growth rate constant, k, also decreased as a function of the Aβ concentration. At 1 µM Aβ1-42, k decreased from ~0.45 h^−1^ (Aβ alone) to ~0.18–0.14 h^−1^, while at 10 µM Aβ1-42, k decreased from ~2.5 h^−1^ to ~1.71 h^−1^ with increasing tau seed concentrations (Figure 1C,I). In both cases, this reduction reflects rapid early monomer consumption and distribution of the elongation of fibrillary ends, resulting in a broader, less steep growth phase. In contrast, at 20 µM Aβ1-42, where monomer availability is high, k increased markedly from ~1.35 h^−1^ (Aβ alone) to ~4.0–4.1 h^−1^ with 10–20 µM tau seeds (Figure 1O). This trend suggests that under monomer-abundant conditions, the presence of a large number of growth-competent fibril surfaces drives a steep early growth phase. Thus, our results reveal that tau seeds consistently accelerate Aβ aggregation kinetics (i.e., lower t_50_), while the apparent growth rate constant k depends on the balance between monomer availability and seed density, as k was found to decrease under monomer-limited conditions (1 and 10 µM Aβ1-42) but increased with its abundance (20 µM Aβ1-42). Collectively, these results suggest that tau seeds promote Aβ assembly primarily through elongation- and secondary nucleation-dominated mechanisms, which is consistent with their templating role in seeding-dependent aggregation.

Augmented Aβ1-42 fibrilization in the presence of 0N4R tau seeds was validated by dynamic light scattering (DLS) analysis, which revealed a corresponding increase in Aβ aggregate size from ~200–600 nm in hydrodynamic diameter in the absence of tau seeds to ~1000–1600 nm in the presence of 20 µM tau seeds (Figure 1D,E,J,K,P,Q). This was further confirmed by fluorescence (Supplementary Figure S1A–C) and transmission electron microscopy (TEM) (Figure 1F,L,R) imaging, which showed a higher abundance of Aβ fibrillar structures with increasing 0N4R tau seed concentrations. Taken together, these findings indicate that 0N4R tau seeds significantly enhance Aβ1-42 aggregation in a concentration-dependent manner, possibly by accelerating secondary and elongation phases. Based on these data, we used 10 µM 0N4R tau seeds + 10 µM Aβ1-42 (1:1 ratio) for subsequent experiments involving PLGA nanoparticles.

### 2.2. Effect of PLGA on 0N4R Tau-Seed-Induced Aβ1-42 Aggregation

**Figure 2:**
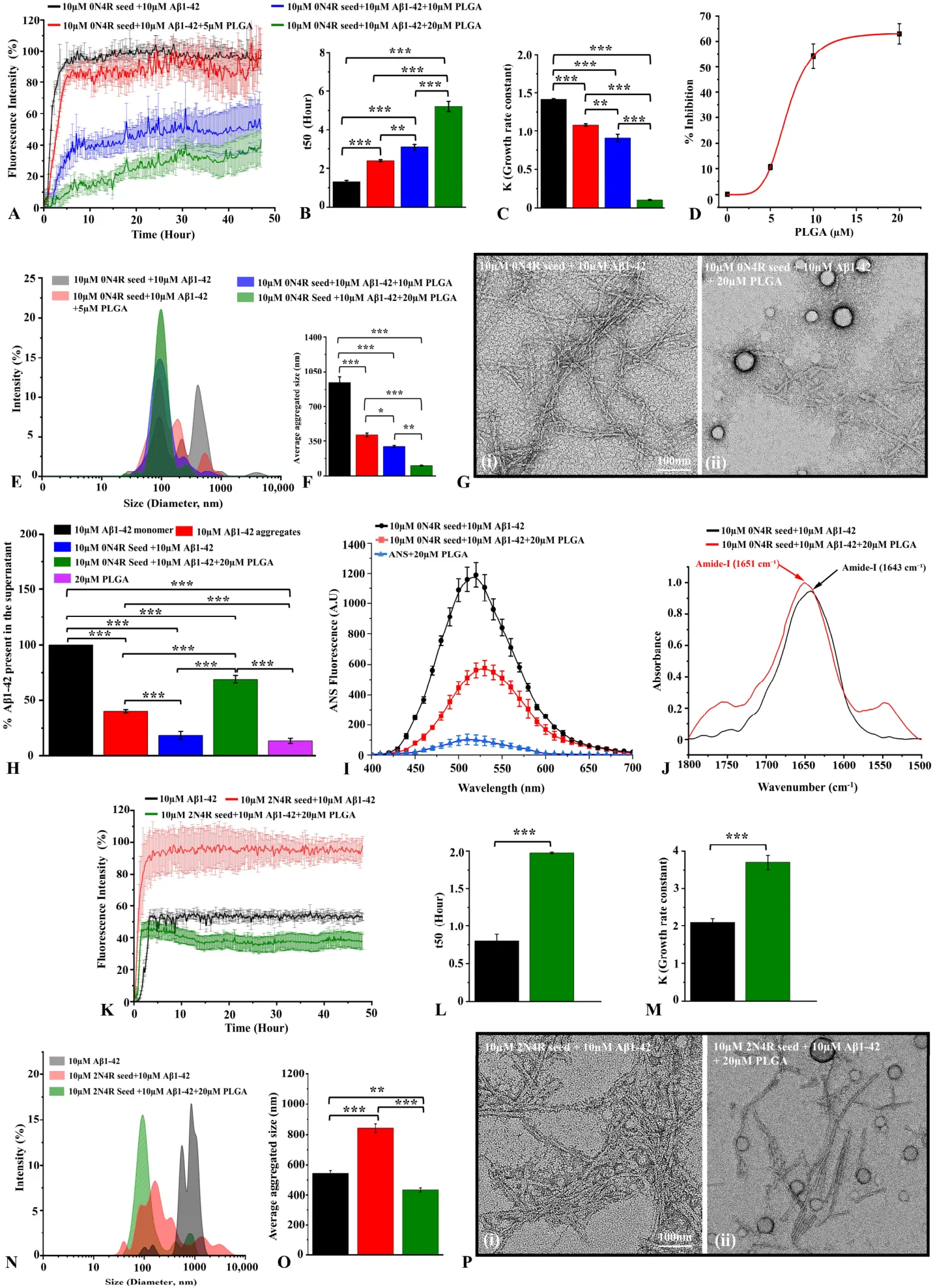
Attenuation of tau-seed-induced Aβ1-42 aggregation by native PLGA. (**A**–**G**): Native PLGA dose dependently (5–20 µM) attenuated 10 µM 0N4R tau-seed-induced aggregation of 10 µM Aβ1-42 over 48 h at 37 °C, as revealed by ThT kinetics assays (**A**–**D**) and the respective DLS analysis (**E**,**F**), as well as TEM (**G**) images acquired after 48 h incubation. Note the inhibition profile and the calculated IC_50_ of native PLGA (**D**). DLS analysis (**E**,**F**) reveals a decrease in the diameter of Aβ1-42 in the presence of increasing concentrations of PLGA compared to control Aβ1-42, as depicted by size distribution curves (**E**) and the histogram (**F**) representing average aggregate size. TEM (**G**) imaging shows the formation of short broken fibrils or smaller-order aggregates in the presence of PLGA (**Gii**) compared to higher-order fibrillar aggregates found in tau-seed-induced Aβ1-42 (**Gi**). (**H**) NanoDrop-based solubility analysis of Aβ1-42 after 48 h incubation depicting that tau seeds markedly reduced the Aβ fraction present in the supernatant compared with Aβ alone, whereas 20 µM PLGA substantially increased the soluble fraction in the presence of tau seeds. (I) ANS fluorescence emission spectra of tau-seed-induced Aβ1-42 aggregates in the absence (black) and presence (red) of 20 µM PLGA. Note that PLGA decreased ANS fluorescence intensity, consistent with reduced exposure of hydrophobic surfaces in the aggregate population. Also, note that PLGA + ANS exhibited a relatively low fluorescence signal (blue) compared with tau-seed-induced Aβ1-42 aggregates. (**J**) FTIR spectra of tau-seed-induced Aβ1-42 aggregates recorded in the absence (black) and presence (red) of 20 µM native PLGA. The amide I maximum centered at ~1643 cm^−1^ in the tau seed condition shifted to a higher frequency, i.e., ~1651 cm^−1^, upon PLGA treatment, indicating altered aggregate secondary structure/packing. (**K**–**P**) Native PLGA attenuated 2N4R tau-seed-induced Aβ1-42 aggregation, as revealed by ThT kinetic assays (**K**–**M**), DLS analysis (**N**,**O**) and TEM (**P**) imaging after 48 h incubation. All ThT kinetic graphs represent mean ± SEM of three separate experiments, each performed with six replicates for each condition. n.s. represents non-significant. * *p* < 0.05, ** *p* < 0.01, *** *p* < 0.001.

Prior to assessing the effects of native PLGA on tau-seed-induced Aβ aggregation, we revealed that native PLGA nanoparticles used in the study displayed a diameter of ~100 nm with spheroidal morphology, as observed with DLS and TEM analysis, respectively (Supplementary Figure S3A,B). Consequently, we evaluated the effects of 5, 10 and 20 µM native PLGA on 10 µM 0N4R tau-seed-induced aggregation of 10 µM Aβ1-42 using a ThT kinetic assay. Our results indicated that native PLGA dose dependently inhibited Aβ aggregation induced by tau seeds over a 48 h incubation at 37 °C, as reflected by progressively lower ThT fluorescence intensities compared to untreated controls (Figure 2A). At a 5 µM concentration, PLGA displayed only a modest inhibitory effect, whereas 10 and 20 µM PLGA markedly attenuated Aβ aggregation compared with the untreated samples. Suppression of Aβ aggregation over 48 h of incubation with 5, 10 and 20 µM native PLGA was approximately 10.2%, 42.7%, and 52.4%, respectively (Figure 2A). Normalized ThT kinetic traces were fitted using a logistic IV function to determine the apparent rate constant (k) and half-time (t_50_). The t_50_ increased from 1.3 h in the absence of PLGA to 2.41, 3.10 and 5.20 h in the presence of 5, 10, and 20 µM PLGA, respectively, while k decreased simultaneously from 1.41 to 1.08, 0.91 and 0.1 h^−1^ across the same concentration range (Figure 2B,C; Supplementary Figure S2D). The progressive increase in t_50_ and decrease in k indicate that PLGA slows the growth and elongation phases of Aβ aggregation, which is consistent with the reduced seeding efficiency. These results suggest that native PLGA interferes with tau seed-mediated elongation or secondary nucleation, but it did not allow us to distinguish the precise underlying mechanisms involved. Nonlinear fitting of the inhibition profile yielded an apparent IC_50_ of ~6.9 µM and a fitted maximal inhibitory effect (Emax) of approximately 63% (Figure 2D). In this analysis, IC_50_ represents the concentration of PLGA producing 50% of the fitted maximal inhibitory effect rather than 50% absolute inhibition of Aβ1-42 aggregation. Since the fitted Emax remained substantially below 100%, the data are consistent with a partial inhibitory profile of native PLGA under the present experimental conditions. The inhibitory effect of PLGA on Aβ aggregation was subsequently validated by DLS analysis, which showed a decrease in the hydrodynamic diameter of Aβ1-42 aggregates from ~1000 nm (i.e., seeded control) to ~100–200 nm with increasing PLGA concentrations (Figure 2E,F). Fluorescence imaging confirmed decreased presence of Aβ fibrillar entities with increasing PLGA concentration (Supplementary Figure S4A). TEM analysis of Aβ samples after 48 h of incubation also revealed that tau-seed-induced Aβ fibrils in the absence of PLGA, as expected, appeared as long fibrils, whereas the PLGA-treated samples displayed mostly as short, twisted, and fragmented fibrils (Figure 2G). These morphological changes indicate that PLGA likely interacts with fibrillary surfaces, partially capping elongation sites and diverting growth toward less ordered structures.

To further validate our results, we determined the soluble Aβ fraction in tau-seed-induced Aβ1-42 aggregation in the presence and absence of native PLGA nanoparticles using a NanoDrop-based assay. As shown in Figure 2H, the Aβ fraction remaining in the supernatant decreased significantly from ~40% to ~18% in the presence of tau seeds, consistent with increased formation of sedimentable Aβ aggregates. Interestingly, the presence of 20 µM native PLGA substantially enhanced the Aβ fraction in the supernatant to ~70%, suggesting attenuation of the formation of tau-seed-induced Aβ aggregates. Subsequently, to assess whether changes in surface hydrophobicity were associated with the PLGA-mediated inhibition of Aβ aggregates, 8-Anilino-1-Naphthalene Sulfonate (ANS) fluorescence measurements were performed on tau-seed-induced Aβ1-42 aggregation in the presence or absence of 20 µM native PLGA (Figure 2I). Strong ANS emission was evident in tau-seed-induced Aβ aggregation, suggesting extensive exposure of hydrophobic surfaces, characteristic of β-sheet-rich conformational changes. Interestingly, native PLGA reduced ANS fluorescence intensity by ~50%, highlighting diminished β-sheet conformational changes, leading to the formation of less-ordered compact species. As for the control, we performed ANS binding with native PLGA in the absence of Aβ and/or tau, which showed a relatively low fluorescence signal compared with tau-seed-induced Aβ1-42 aggregates (Figure 2I). This observation was further supported by FTIR spectroscopy, which showed that the amide-I band of tau-seed-induced Aβ1-42 aggregates shifted from ~1643 cm^−1^ to ~1651 cm^−1^ following PLGA treatment. It is of interest to note that the PLGA-alone absorbance spectrum was subtracted prior to normalization and analysis, indicating that the observed shift was not attributable to the intrinsic spectral contribution of PLGA (Figure 2J). Taken together, these results indicate that native PLGA likely attenuates tau-seed-induced Aβ1-42 aggregation by limiting the formation of highly ordered hydrophobic β-sheet-rich assemblies. Consequently, we demonstrated that 10 µM 2N4R tau-seed-induced Aβ1-42 aggregation could also be attenuated by 20 µM native PLGA over 48 h of incubation, as observed in our ThT kinetic assay (Figure 2K–M; Supplementary Figure S2E), DLS (Figure 2N,O), fluorescence imaging (Supplementary Figure S4B) and TEM analysis (Figure 2P). The kinetic fitting of the 2N4R tau-seed-induced Aβ1-42 aggregation curves showed that PLGA increased t_50_ from 0.8 to 1.98 h, indicating delayed aggregation. The apparent rate constant (k) increased from 2.08 to 3.70 h^−1^, reflecting a steeper growth phase following the delayed onset of aggregation (Figure 2L,M; Supplementary Figure S2E). These data indicate that native PLGA nanoparticles can effectively attenuate tau-seed-induced Aβ1-42 aggregation through mechanisms that are consistent with seed passivation and interference with elongation and secondary nucleation.

### 2.3. Effect of PLGA on Tau Monomeric vs. Seed-Induced Aβ1-42 Aggregation

**Figure 3:**
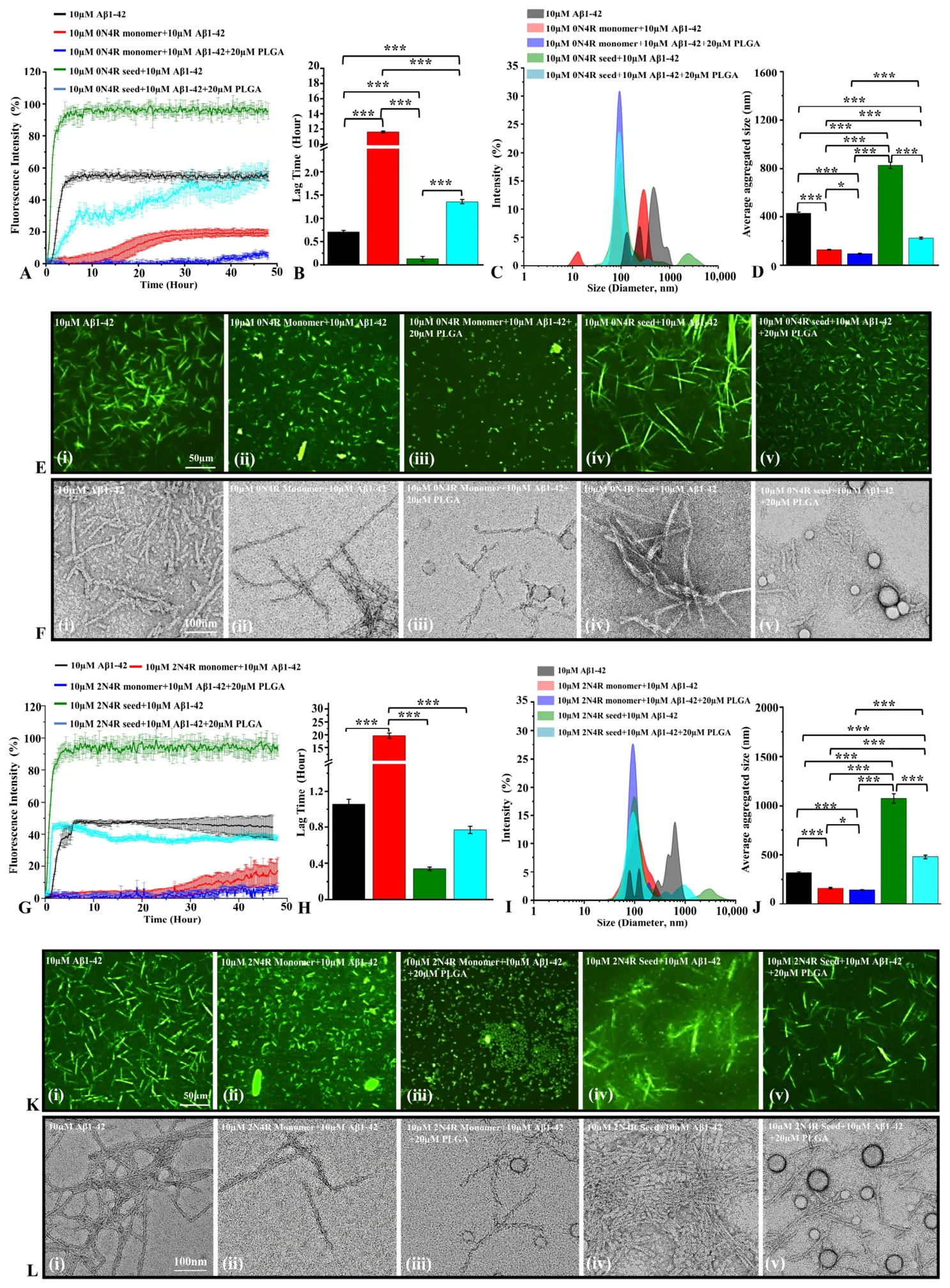
Tau monomer vs. tau seeds on Aβ1-42 aggregation and modulation by PLGA. (**A**–**F**): ThT kinetic assays showing aggregation curves (**A**,**B**), and the corresponding DLS analysis (**C**,**D**), fluorescence (**E**), as well as TEM (**F**) images of 10 µM Aβ1-42 in the absence or in the presence of 10 µM 0N4R tau monomer or 10 µM 0N4R tau seeds with or without 20 µM PLGA, over 48 h at 37 °C. Note that tau seeds, as observed earlier, enhanced Aβ1-42 aggregation, whereas monomeric tau markedly decreased Aβ1-42 aggregation with extended lag phase, as observed in ThT kinetic assays (**A**,**B**), DLS analysis (**C**,**D**), fluorescence (**E**) and TEM (**F**) images. The presence of native PLGA attenuated aggregation of Aβ1-42, as observed in our ThT kinetic assay (**A**,**B**), DLS analysis (**C**,**D**), fluorescence (**Eiii**,**Ev**) and TEM (**Fiii**,**Fv**) imaging. (**G**–**L**): ThT kinetic assays showing aggregation curves (**G**,**H**), and the corresponding DLS analysis (**I**,**J**), fluorescence (**K**) and TEM (**L**) images of 10 µM Aβ1-42 in the absence or in the presence of 10 µM 2N4R tau monomer or 10 µM 2N4R tau seeds with or without 20 µM PLGA, over 48 h at 37 °C. Note that 2N4R tau seed enhanced Aβ1-42 aggregation, whereas monomeric tau decreased Aβ1-42 aggregation with extended lag phase as observed in ThT kinetic assays (**G**,**H**), DLS analysis (**I**,**J**), fluorescence (**K**) and TEM (**L**) images. The presence of native PLGA attenuated aggregation of Aβ1-42, as observed in our ThT kinetic assay (**G**,**H**), DLS analysis (**I**,**J**), fluorescence (**Kiii**,**Kv**) and TEM (**Liii**,**Lv**) imaging. All ThT kinetic graphs represent mean ± SEM of three separate experiments, each performed with six replicates for each condition. * *p* < 0.05, *** *p* < 0.001.

To determine whether monomeric tau can induce Aβ1-42 aggregation, as observed with tau seeds (Supplementary Figure S3C–E), we first performed ThT kinetic assay by incubating 10 µM Aβ1-42 in the presence or absence of 0N4R tau monomers, as well as tau seeds at 37 °C for 48 h. Interestingly, while tau seeds, as observed earlier, significantly enhanced Aβ1-42 aggregation, monomeric tau markedly decreased the aggregation kinetics of Aβ1-42 with an extended lag phase (Figure 3A,B). These contrasting results were corroborated using DLS, fluorescence imaging, and TEM study. DLS analysis showed that 0N4R tau monomer-treated samples formed smaller Aβ aggregates, whereas tau seed-treated counterparts formed larger Aβ aggregates compared to aggregates formed in the absence of tau protein (Figure 3C,D). Additionally, fluorescence imaging (Figure 3E) and TEM (Figure 3F) revealed that Aβ1-42 samples incubated with 0N4R tau monomers formed fewer fibrillary structures than those treated with 0N4R tau seeds. Consequently, we assessed the effects of native PLGA on 0N4R tau monomeric and tau-seed-induced Aβ1-42 aggregation. As expected, native PLGA nanoparticles decreased further Aβ1-42 aggregation observed in the presence of monomeric tau compared to the corresponding monomer + Aβ1-42 samples (Figure 3A,B). DLS analysis (Figure 3C,D), fluorescence imaging (Figure 3E), and TEM (Figure 3F) supported these findings, showing that PLGA-treated samples produced fewer and smaller Aβ aggregates than the untreated samples. To further validate these results, we subsequently performed ThT kinetic assays using 10 µM Aβ1-42 in the presence or absence of 2N4R tau monomers, as well as tau seeds, over a 48 h period at 37 °C. A similar effect was observed with 2N4R tau, where 2N4R monomeric tau substantially reduced Aβ1-42 aggregation and 2N4R tau seeds markedly enhanced Aβ aggregation (Figure 3G,H). Additionally, native PLGA treatment decreased further Aβ1-42 aggregation observed in the presence of both the monomeric tau as well as tau seeds, though the effect is more prominent in the presence of tau seeds than that observed with monomeric tau (Figure 3G,H). This was confirmed using DLS analysis (Figure 3I,J), fluorescence imaging (Figure 3K), and TEM (Figure 3L). Taken together, these results suggest that tau monomers, in contrast to tau seeds, decrease Aβ1-42 aggregation, and native PLGA was found to reduce further Aβ1-42 aggregation observed in the presence of monomeric tau as well as tau seeds.

### 2.4. Dot-Blot Analysis of PLGA Effects on Tau Conformer-Mediated Aβ1-42 Aggregation

**Figure 4:**
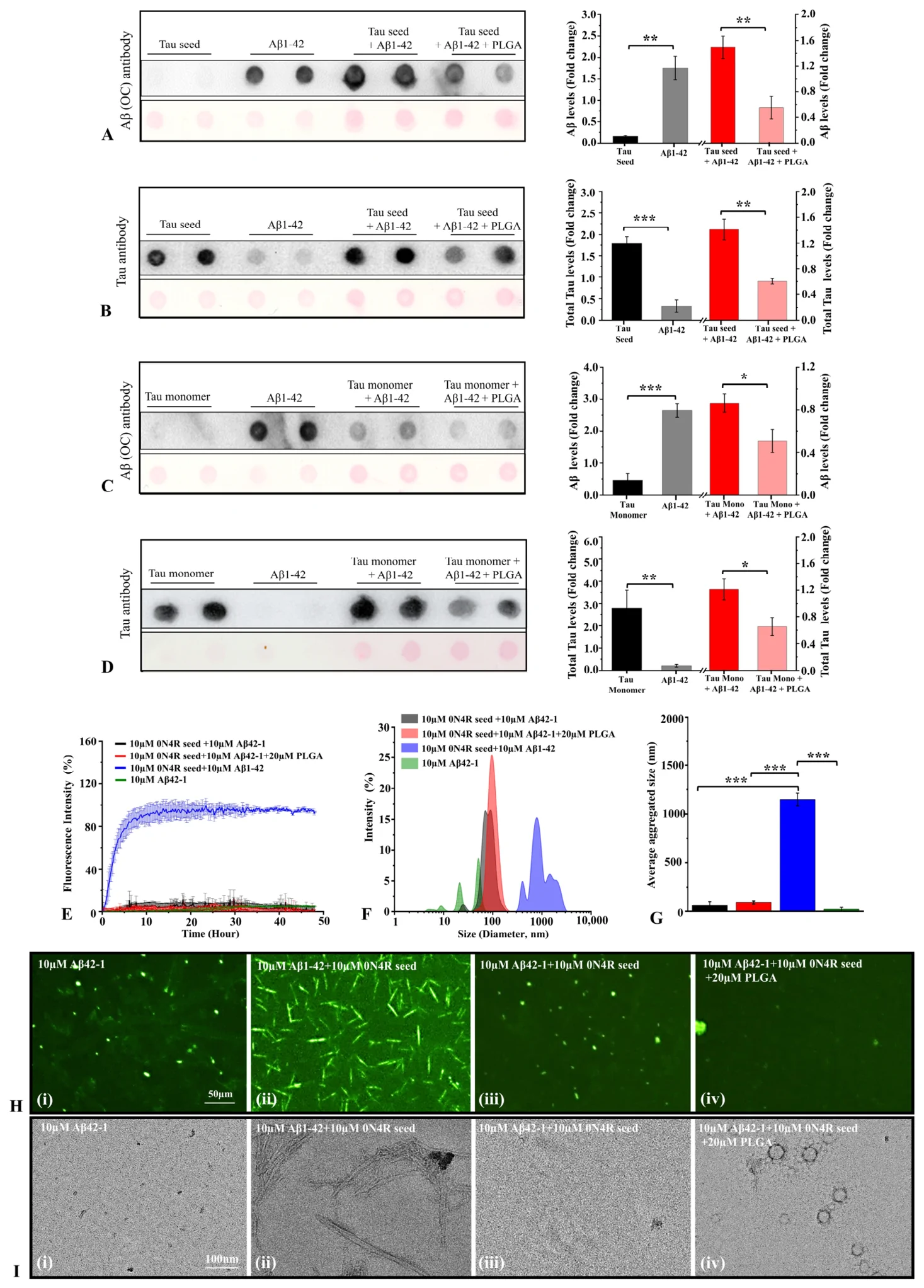
Specificity of 0N4R tau-seed-induced Aβ1-42 aggregation and native PLGA. (**A**–**D**): Dot-blots and corresponding quantifications showing the interactions of tau-seed- (**A**,**B**) and tau-monomer- (**C**,**D**) induced Aβ1-42 aggregation in the absence and presence of 20 µM PLGA, as detected using Aβ fibrillar-specific OC antibody (**A**,**C**) and total tau Tau5 antibody (**B**,**D**). The corresponding dot-blots were stained with Ponceau to depict loading of protein samples. Note that tau seeds significantly enhanced aggregation of Aβ1-42, which is decreased in the presence of native PLGA (**A**,**B**). Tau monomer, on the other hand, reduces aggregation of Aβ1-42 compared to Aβ1-42 alone and this is found to be decreased further in the presence of native PLGA nanoparticles (**C**,**D**). (**E**–**I**): ThT kinetic assays showing aggregation curves (**E**), and the corresponding DLS analysis (**F**,**G**), fluorescence (**H**) and TEM (**I**) images of 10 µM Aβ1-42 and the reverse sequence control 10 µM Aβ42-1. Note that tau seeds, unlike Aβ1-42, did not alter aggregation of Aβ42-1. This is evident in our ThT kinetic assay (**E**), DLS analysis (**F**,**G**), fluorescence (**Hi**,**Hii**,**Hiii**) or TEM (**Ii**,**Iii**,**Iiii**) imaging. Note that native PLGA also did not affect the aggregation of Aβ42-1 as evident in our ThT kinetic assays (**E**), DLS (**F**,**G**), fluorescence (**Hiii**,**Hiv**) or TEM (**Iiii**,**Iiv**) analysis. All ThT kinetic graphs represent mean ± SEM of three separate experiments, each performed with six replicates for each condition. * *p* < 0.05, ** *p* < 0.01, *** *p* < 0.001.

Consistent with our ThT kinetic assay and structural studies, our dot-blot analysis revealed that tau seed-enhanced Aβ1-42 aggregation is significantly attenuated following treatment with native PLGA, as evidenced by the immunoreactive signals detected using Aβ fibrillar-specific OC and tau Tau5 antibodies (Figure 4A,B). Conversely, monomeric tau, which significantly decreases Aβ1-42 aggregation in the absence and presence of PLGA, was also apparent in our dot-blot analysis (Figure 4C,D). Ponceau S staining confirmed comparable protein loading across different experimental conditions. Collectively, our dot-blot results confirm that tau seeds and monomeric tau differentially regulate Aβ1-42 aggregation and that both these effects are mitigated by native PLGA.

### 2.5. Specificity of Aβ Aggregation Induced by 0N4R Tau Seed

To determine the specificity of Aβ1-42 aggregation induced by tau seeds, we evaluated the aggregation of 10 µM Aβ1-42 and Aβ42-1 (i.e., Aβ42-1, a negative control peptide) in the presence or absence of 10 µM 0N4R tau seed using a ThT kinetic assay (Figure 4E), DLS analysis (Figure 4F,G), fluorescence imaging (Figure 4H), and TEM (Figure 4I). Unlike normal Aβ1-42, tau seeds did not induce aggregation of Aβ42-1, validating the specificity of this effect (Figure 4E–I). Additionally, native PLGA, as expected, attenuated aggregation of Aβ1-42 but did not influence aggregation kinetics of Aβ42-1 over 48 h at 37 °C (Figure 4E–I).

### 2.6. Effects of PLGA on the Formation of Tau Seeds and Its Influence on Aβ1-42 Aggregation

**Figure 5:**
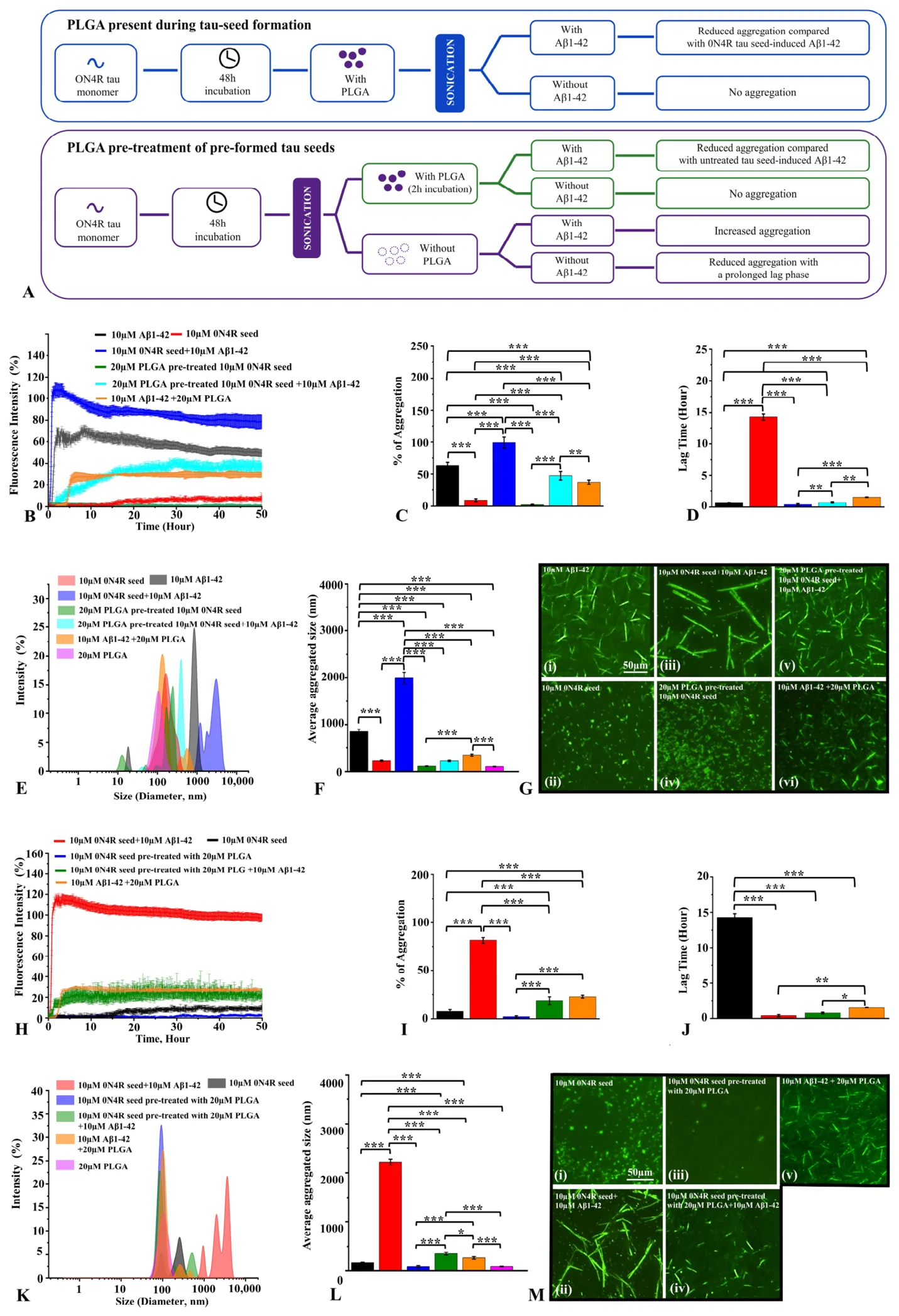
Native PLGA, tau aggregation/tau seed formation and Aβ1-42 aggregation. (**A**): Schematic representation used to examine whether the interaction between native PLGA and tau can influence subsequent Aβ1-42 aggregation in the following two experimental paradigms; top panel represents the presence of native PLGA during the formation of tau seeds and its influence on Aβ1-42 aggregation and bottom panel represents pretreatment of tau seeds with PLGA prior to performing Aβ aggregation analysis. B-G: ThT kinetic profiles of 10 µM Aβ1-42, 10 µM Aβ1-42 + 20 µM PLGA, 10 µM 0N4R tau seeds, 10 µM tau seed + 10 µM Aβ1-42, 20 µM PLGA-treated tau seeds and 20 µM PLGA-treated tau seed + 10 µM Aβ1-42, over 48 h at 37 °C (**B**–**D**) and the corresponding DLS analysis (**E**,**F**) as well as fluorescence imaging (**G**) showing the effect of PLGA on tau seed formation (**Giv**) and its influence on Aβ1-42 aggregation (**Gv**). Note that PLGA-untreated tau seeds displayed a robust Aβ1-42 aggregation (**Giii**) compared to treated tau seeds (**Gv**), while Aβ1-42 aggregation exhibited a similar profile in the presence of PLGA-treated tau seeds (**Gv**) as observed with PLGA alone (**Gvi**). H-M: ThT kinetic profiles of 10 µM 0N4R tau seeds, 10 µM tau seed + 10 µM Aβ1-42, 20 µM PLGA-pretreated tau seeds, 20 µM PLGA-pretreated tau seed + 10 µM Aβ1-42 and 10 µM Aβ1-42 + 20 µM PLGA over 48 h at 37 °C (**H**–**J**) and the corresponding DLS analysis (**K**,**L**) as well as fluorescence imaging (**M**) showing the effect of PLGA-pretreated tau seeds on Aβ1-42 aggregation. Note that PLGA-untreated tau seeds triggered a strong aggregation of Aβ1-42 (**Mii**) compared to PLGA-treated tau seeds (**Miv**), while aggregation of Aβ1-42 exhibited a similar profile in the presence of PLGA-treated tau seeds (**Miv**), as observed with PLGA alone (**Mv**). All ThT kinetic graphs represent mean ± SEM of three separate experiments, each performed with six replicates for each condition. * *p* < 0.05, ** *p* < 0.01, *** *p* < 0.001.

To determine whether native PLGA regulates seeding competence of tau fibrils and its ability to influence Aβ1-42 aggregation, 10 µM monomeric 0N4R tau was incubated with heparin for 48 h at 37 °C, either alone (untreated tau seeds) or together with 20 µM native PLGA (PLGA-treated tau seeds), followed by probe sonication (Figure 5A). These two tau seed preparations were used in parallel to induce Aβ1-42 aggregation in the ThT kinetic assays. As for the controls, we used Aβ1-42 alone, Aβ1-42 + PLGA, or tau seeds without PLGA. Our ThT fluorescence kinetic analysis revealed that untreated tau seeds exhibited robust Aβ1-42 aggregation compared with PLGA-treated tau seeds (Figure 5B–D). Interestingly, Aβ1-42 in the presence of PLGA-treated tau seeds displayed aggregation kinetics similar to those observed with Aβ1-42 + PLGA alone (Figure 5B–D). Interestingly, PLGA-treated tau seeds showed a significant decrease in the ThT signal compared to untreated tau seeds, indicating impaired formation of tau seeds (Figure 5B–D). This observation is supported by our DLS analysis, which showed that the hydrodynamic diameters of various aggregates used in different experimental conditions were as follows: Aβ1-42 alone ~800–900 nm, tau seeds without PLGA treatment ~150–200 nm, Aβ1-42 + tau seeds without PLGA treatment ~1900–2000 nm, tau seeds with PLGA treatment ~100–150 nm, and Aβ1-42 + tau seeds with PLGA treatment ~200–250 nm (Figure 5E,F). These results were complemented by fluorescence imaging of various experimental samples obtained following ThT kinetic analysis (Figure 5G). Collectively, these data indicate that tau seeds formed in the presence of PLGA are structurally and functionally distinct from those formed in the absence of PLGA and reduce the ThT signal, smaller particle size, and diminished ability to promote Aβ1-42 aggregation. These findings indicate that the presence of PLGA during tau aggregation interferes with the formation of tau seeds and substantially attenuates their subsequent heterologous seeding activity toward Aβ1-42.

### 2.7. Effects of PLGA Pretreatment on Tau Seeds and Their Influence on Aβ1-42 Aggregation

To determine whether PLGA directly alters the seeding competence of tau fibrils, pre-formed 0N4R tau seeds were incubated with or without 20 µM PLGA for 2 h prior to determining their effects on the aggregation of 10 µM Aβ1-42 (Figure 5A). Our ThT kinetic analysis showed that untreated PLGA tau seeds markedly increased Aβ1-42 aggregation, whereas PLGA-treated tau seeds exhibited substantially reduced Aβ1-42 aggregation (Figure 5H–J). Consistent with ThT kinetics, DLS analysis revealed that untreated tau seeds + Aβ1-42 produced large aggregates (hydrodynamic diameters ~2200 nm) compared to those observed in PLGA-treated tau seeds + Aβ1-42 (hydrodynamic diameters ~350 nm) (Figure 5K,L). As expected, tau seeds alone displayed smaller aggregates (hydrodynamic diameters ~150–200 nm), which were further reduced following PLGA treatment (hydrodynamic diameters ~80–100 nm). This observation was supported by the fluorescence imaging results (Figure 5M) of various samples obtained by ThT kinetic assays. As for the control, we showed that 20 µM of native PLGA alone did not alter ThT fluorescence intensity over 48 h of incubation, indicating PLGA itself does not affect ThT fluorescence under our experimental conditions (Supplementary Figure S5A). Additionally, to examine the effect of PLGA on tau seed activity, pre-formed 0N4R tau seeds were incubated with 20 µM PLGA for 2 h and centrifuged at 14,000 rpm for 90 min, and the supernatant was subsequently incubated with 10 µM Aβ1-42. The supernatant showed higher ThT fluorescence and significantly larger Aβ aggregates compared to the corresponding PLGA-pretreated tau seed samples without centrifugation (Supplementary Figure S5B–D). Collectively, these results indicate that PLGA treatment of tau seeds markedly diminishes their ability to trigger Aβ1-42 aggregation, possibly because of the reduced availability of growth-competent seed surfaces.

## 3. Discussion

The present study revealed that tau seeds, in contrast to monomeric tau, can markedly enhance Aβ1-42 aggregation, which is attenuated in the presence of native PLGA nanoparticles. This is supported by results showing that: (i) pre-formed 0N4R and 2N4R tau seeds dose dependently increased Aβ1-42 aggregation, as evident by ThT kinetic assay, DLS, ANS, fluorescence imaging, and TEM analysis; (ii) native PLGA nanoparticles with increasing concentrations attenuated both 0N4R and 2N4R tau-seed-induced Aβ1-42 aggregation; (iii) monomeric 0N4R and 2N4R tau decreased Aβ1-42 aggregation, and the effect was reduced further by native PLGA; and iv) tau aggregation and formation of tau seeds in the presence of PLGA also attenuated Aβ1-42 fibrillization. Collectively, these results suggest that native PLGA, without conjugation with any drug or therapeutic agent, can suppress tau-seed-induced aggregation of Aβ1-42—an Aβ isoform that plays a critical role in the degeneration of neurons and the development of AD pathology.

Under normal conditions, monomeric Aβ exists as an unstructured random-coil, but its fibrillization due to increased levels driven by overproduction and/or reduced clearance results in a cross-β structure characterized by β-strand stacking perpendicular to the long fiber axis [37,38,39]. Intramolecular hydrophobic interactions within Aβ residues 17–24 (KLVFFAED) are critical for stabilizing the cross-β-sheet structure, while the C-terminal domain promotes protofilament formation by generating a hydrophobic core involving Ile41 and Val39 of the adjacent fibril. Additional residues, including His14, Gln15, Ala30, Ile31, Met35 and Val36, also contribute to Aβ oligomerization and fibril formation [39,40,41]. Monomeric tau, unlike Aβ peptide, is an unstructured cytoplasmic protein whose primary physiological role is microtubule stabilization, without any inherent tendency to aggregate [42]. However, post-translational modifications of the tau protein such as phosphorylation, acetylation, and glycation alter its microtubule-binding domains (MTBDs), triggering the pathological conversion of the monomer into fibrils. This cascade begins with tau dimerization, driven either by intermolecular disulfide cross-linking between cysteine residues Cys291 (R2 domain) and Cys322 (R3 domain), or by electrostatic attractions between the positively charged proline-rich region and the negatively charged N-terminal domain and MTBDs. These dimers subsequently assemble via aggregation-prone hexapeptide motifs (VQIVYK and VQIINK) within the MTBDs to generate oligomers. Over time, these oligomers elongate into ordered β-sheet fibrils, ultimately yielding paired helical filaments (PHFs), the fundamental structural units of neurofibrillary tangles (NFTs) [43]. The recombinant tau, unlike post-translationally modified tau, does not aggregate spontaneously due to its hydrophilic nature [42]. Thus, tau aggregation under in vitro conditions is usually studied by the addition of polyanionic factors such as heparin, which initiate aggregation by triggering a conformational change in the hexapeptide motifs in MTBDs [44].

Earlier studies have shown that Aβ seeds can initiate aggregation and propagation of tau pathology [45,46,47,48]. Although the mechanism remains unclear, it has been suggested that pre-aggregated Aβ with stackable β-strands can increase the hydrophobic surface area, facilitating template-assisted growth of tau protein [49]. The present study, as a complement to the Aβ-induced tau aggregation, reveals that pre-formed 0N4R and 2N4R tau seeds can dose dependently enhance Aβ1-42 aggregation. This is apparent by ThT kinetic assays, fluorescence imaging, TEM and DLS analyses showing the generation of Aβ aggregates with a higher hydrodynamic radius. Our kinetic fitting analysis indicates that the presence of tau seeds shortened the onset of Aβ1-42 aggregation, most likely by bypassing the primary nucleation phase. The apparent growth rate constant, on the other hand, was found to vary with Aβ concentration, i.e., decreasing under monomer-limited conditions and rising with the enhanced monomer availability, suggesting a shift in the growth process. Collectively, this profile supports elongation- and secondary-nucleation-driven Aβ1-42 aggregation in the presence of tau seeds, but it needs to be validated in future studies. Several agents including non-chaperones (e.g., albumin), chaperones (e.g., DNAJB6) and amyloid-related proteins (α-synuclein) have been shown to enhance or decrease Aβ fibrillization [50,51,52,53]. Based on these results, it can be interpreted that 0N4R and 2N4R tau seeds, by regulating the surface/binding affinity of the monomeric Aβ or Aβ species formed during primary/secondary nucleation, can lead to enhanced Aβ fibrilization, as observed in the present study. This is supported by four distinct lines of evidence: (i) the Aβ core region (16KLVFFA21) and its C-terminal residues (37GGVVIA42 exclusive to Aβ1-42) have been shown to interact with the aggregation-prone VQIINK and VQIVYK motifs of tau [17]; (ii) N-acetylated and C-amidated hexapeptide AcPHF6 (Ac-VQIVYK-NH2) derived from the tau aggregation-prone VQIVYK motif has been shown to accelerate aggregation kinetics resulting in an enhanced Aβ fibrillogenesis [19]; (iii) tau has been shown to enhance Aβ aggregation by co-aggregating with it [54]; and (iv) recent data show that tau fragments spanning 297–391 residues, which are capable of spontaneously assembling into amyloids with PHF, can promote heterotypic Aβ1-42 primary nucleation via direct interaction, leading to enhanced Aβ fibrillization [55].

Interestingly, monomeric 0N4R and 2N4R tau, unlike tau seeds, were found to attenuate spontaneous aggregation of 10 µM Aβ1-42 by delaying the lag phase and reducing the growth phase. This is consistent with earlier studies which showed that tau monomer can inhibit Aβ1-42 fibrillization by interfering with the transition of unstructured Aβ monomers into β-sheet-rich structures and by the suppressing elongation/secondary fibrillation process [20,56]. A subsequent study further revealed that the aggregation-prone tau repeats “VQIINK” and “VQIVYK”, by interacting with the hydrophobic regions of Aβ, can inhibit not only Aβ fibrillogenesis but also can promote dissociation of pre-formed Aβ fibrils under in vitro conditions [57]. These results, taken together, highlight that the structural state of tau (monomer vs. seeds), as observed in the present study, may have a differential role in determining whether to inhibit or promote Aβ assembly.

Although Aβ dysfunction is known to precede tau pathology, it is believed that the phosphorylation and aggregation of tau protein via intertwined interplay can also influence Aβ pathology in AD brains [3,58,59]. This is supported by data showing that (i) the presence of NFTs appears prior to Aβ-containing plaques in some AD cases [60,61], (ii) monomeric tau can bind to both monomeric and fibrillar Aβ [56], (iii) the presence of tau is essential for mediating Aβ toxicity [14], and (iv) reducing endogenous tau mitigates Aβ-induced memory deficits and decreases early mortality in a transgenic mouse model of AD [62]. This interaction is supported by the detection of Aβ-bound tau complexes in AD brains [17,63]. Thus, molecules that target different facets of both Aβ and tau may offer a better prospect to treat AD than those targeting either protein independently.

A number of earlier studies have shown that various nanoparticles functionalized with phytochemicals (resveratrol and curcumin), hormones (estradiol and melatonin), antibodies, and drugs (memantine and selegiline) can interfere with Aβ and/or tau aggregation/toxicity [64,65,66,67,68]. More recently it has been shown that a chiral nanostructure based on -copper organic helics [D(L)-Cu-SMOHs] not only exhibits high Aβ inhibition efficiency but also chirality-discrepant performances, unlike other functionalized inhibitors [69]. Earlier, we reported that native PLGA can inhibit not only spontaneous Aβ and tau aggregation, but also Aβ seed-induced tau aggregation [34,35,36]. The present study extends these results by showing that Aβ1-42 aggregation promoted by tau seeds can also be inhibited by native PLGA. This effect was evident in the presence of both 0N4R and 2N4R tau seeds, suggesting an ability of PLGA to suppress Aβ1-42 aggregation regardless of the tau isoform. Interestingly, Aβ1-42 fibrilization, which was reduced in the presence of monomeric tau compared to Aβ1-42 without tau, was found to be decreased further in the presence of PLGA. Our dose–response analysis revealed an apparent IC_50_ of ~6.9 µM with a fitted Emax of ~63%, indicating a partial inhibitory profile of native PLGA against tau-seed-induced Aβ1-42 aggregation under the given experimental conditions. Accordingly, our kinetic fitting showed that PLGA increased t_50_ while decreasing k, supporting reduced seeding efficiency rather than complete suppression of fibril formation. This was supported by our fluorescence imaging, TEM, and DLS analyses, which showed smaller species of Aβ aggregates. ANS fluorescence intensity also decreased in the presence of PLGA, supporting the existence of less hydrophobic clusters for Aβ aggregation, which was validated by our dot-blot analysis. Additionally, our results revealed that native PLGA, by interfering with the tau fibrilization and/or aggregation component of the tau seed, can reduce Aβ1-42 fibrillogenesis. This could be due to the effects of PLGA on (i) the formation/maturation of β-sheet-rich tau assemblies, (ii) the seeding competence of pre-formed tau fibrils, and (iii) the generation of structurally distinct tau seeds with reduced heterologous seeding competence. These interpretations are somewhat consistent with the mechanism suggesting that PLGA may interact with the surface of pre-existing tau seeds, thereby masking growth-competent interfaces required for Aβ docking and elongation [70]. The specificity of the effect was established by demonstrating that normal Aβ1-42, but not the reverse Aβ42-1 sequence, exhibited accelerated Aβ1-42 aggregation in the presence of tau seeds. Additionally, our centrifugation study revealed that removal of PLGA/tau-associated species from PLGA-pretreated tau seeds partially restored Aβ1-42 aggregation, suggesting that native PLGA interacts with tau-seed-associated species, though it remains unclear whether PLGA decreases the availability of tau seeds or alters their surface properties. Considering the evidence that PLGA can interact with Aβ and tau independently [34,35,36,71], it is possible that the suppression of tau-seed-induced Aβ assembly may be mediated via multiple, non-exclusive mechanisms. First, it may be due to Aβ capping, where PLGA binds to Aβ hydrophobic surfaces and caps elongation-competent ends, thereby slowing the template-assisted growth of Aβ1-42. Second, it may involve PLGA binding to tau protein or tau seeds, thereby decreasing the availability of growth-competent surfaces required for efficient Aβ fibril formation. Third, it may be associated with shared-site blocking, where PLGA may intervene at the common interface where the Aβ core region interacts with the aggregation-prone VQIINK and VQIVYK motifs of tau [17,63] to impede Aβ1-42 fibrilization. These interpretations, however, need to be validated by future experimental studies.

Despite extensive research, no effective treatment is currently available for patients with AD [72,73]. The efficacy of the recently approved disease-modifying Aβ monoclonal antibodies Lecanemab and Donanemab remains controversial [74,75]. Although many strategies targeting Aβ peptides, including structural-based Aβ synthesis/aggregation inhibitors, have been developed over the years, none of them has been clinically approved for the treatment of AD pathology. Considering the complex functional interrelationship between Aβ and tau in the development of AD pathology, it is believed that an approach that can target both Aβ as well as tau simultaneously may provide a more effective treatment strategy for patients with AD [76,77]. The present study showed that native PLGA can suppress tau-seed-induced aggregation of Aβ1-42, but its implications need to be validated using established cellular and/or animal models of AD. Nevertheless, we and others have recently reported that native PLGA nanoparticles can have beneficial effects on cellular/animal models of Parkinson disease [78], amyotrophic lateral sclerosis [79] and AD pathology [33,34,35,36,80]. Additionally, we showed that native PLGA can reduce Aβ seed-induced tau phosphorylation and aggregation [33,36]. These results, together with the evidence that native PLGA can mitigate tau-seed-induced Aβ aggregation, highlight its unique therapeutic potential in the treatment of AD-related pathology, as PLGA can independently target both Aβ and tau proteins and their interactions, which play critical role in the development of AD.

## 4. Materials and Methods

### 4.1. Materials

Recombinant human Aβ1–42 (>97% purity) and Aβ42–1 (>95% purity) were obtained from rPeptide (R&D, Sunnyvale, CA, USA) and Anaspec (Sunnyvale, CA, USA), respectively. PLGA (50:50 resomer, mol. weight: ~30,000) was purchased from Phosphorex (Hopkinton, MA, USA). Human recombinant 0N4R and 2N4R tau proteins were supplied by StressMarq Biosciences Inc. (Victoria, BC, Canada). Hexafluoro-2-propanol (HFIP), ThT, dithiothreitol (DTT) and dimethyl sulfoxide (DMSO) were obtained from Sigma-Aldrich (St. Louis, MO, USA). The bicinchoninic acid (BCA) protein assay kit and enhanced chemiluminescence (ECL) kit were purchased from Thermo Fisher Scientific, Inc. (Nepean, ON, Canada). Heparin sodium salt (tau aggregation inducer) was obtained from Santa Cruz Biotechnology. Carbon-coated 400-mesh copper grids and uranyl acetate stain were purchased from Electron Microscopy Sciences (Fort Washington, PA, USA). Primary antibodies, i.e., Aβ fibrillar-specific OC and total tau Tau5 antibodies, were obtained from EMD Millipore (Burlington, MA, USA). Horseradish peroxidase-conjugated secondary antibodies were obtained from Bio-Rad Laboratories (Hercules, CA, USA). All other standard chemicals were purchased from Sigma-Aldrich or Thermo Fisher Scientific.

### 4.2. Preparation of PLGA Nanoparticles and Tau Seeds

PLGA nanoparticles were prepared according to the manufacturer’s protocol as described previously [34,36]. Briefly, PLGA powder was dissolved in 0.01 M phosphate-buffered saline (PBS, pH 7.4) and sonicated using a probe sonicator (40 pulses at 40% amplitude) before being used in our experiments. The 5, 10 and 20 µM concentrations of PLGA nanoparticles used in the study are based on the PLGA chain molecular weight (i.e., ~30 kDa) and the corresponding mass/volume concentrations are 150 µg/mL, 300 µg/mL and 600 µg/mL. Recombinant human wild-type 0N4R and 2N4R tau proteins (50 μM each) were incubated with heparin sodium salt (0.044 mg/mL) in assay buffer (Dulbecco’s PBS, 2 mM MgCl_2_, 1 mM DTT, pH 7.2) at 37 °C for 48 h to generate tau fibrils. Tau seeds were subsequently prepared by sonicating the fibrils using a probe sonicator (40 pulses at a 30% amplitude). The concentrations of the tau monomer and the resulting sonicated tau seed preparations were determined by BCA assay. The measured concentrations were found to be 523 μg/mL for tau monomer vs. 393 μg/mL for tau seeds, suggesting that the tau seed-to-monomer concentration ratio was approximately 0.75:1 (*w*/*w*).

### 4.3. In Vitro Tau-Seed-Induced Aβ Aggregation

Lyophilized Aβ1-42 and Aβ42-1 peptides were first equilibrated at room temperature for 30 min and then dissolved in HFIP to prepare 1 mM solution. The peptides were subsequently aliquoted, dried using a SpeedVac, and stored at −80 °C until further use. On the day of the experiment, Aβ1-42 was thawed at 4 °C, dissolved in DMSO to 2 mM and then diluted in PBS to 1, 10, and 20 μM. Different concentrations of Aβ1-42 peptide were subsequently incubated at 37 °C for 48 h in the presence or absence of tau seeds (5, 10, or 20 μM) to induce aggregation. Similarly, Aβ42-1 was thawed, dissolved to 2 mM in DMSO, diluted to 10 μM and then incubated with or without 10 μM tau seeds at 37 °C for 48 h. In a parallel set of experiments, 10 μM Aβ1-42 was incubated with 10 μM 0N4R and 2N4R tau monomers as well as tau seeds in the presence or absence of 20 μM of native PLGA. To examine PLGA’s effect, 10 μM Aβ1-42 was incubated with 10 μM tau monomers or seeds, in the presence or absence of different concentrations (5, 10 and 20 μM) of native PLGA at 37 °C for 48 h. Control experiments were performed using 10 μM Aβ42-1 + 10 μM tau seed with or without 20 µM PLGA. Aggregation was monitored by ThT fluorescence assay (20 μM ThT), with readings taken every 15 min for 48 h using a Fluostar Omega (BMG Labtech, Aylesbury, UK) (excitation: 440 nm, emission: 480 nm) [35]. To determine whether native PLGA directly affects ThT fluorescence, 20 μM ThT was incubated with 20 μM native PLGA under the same assay conditions. Additionally, to examine the effect of PLGA on tau seed activity, pre-formed 0N4R tau seeds (10 μM) were first incubated with 20 μM PLGA for 2h and then centrifuged at 14,000 rpm for 90 min. The resulting supernatants were incubated with 10 μM Aβ1-42 for 48 h, and aggregation was evaluated by ThT fluorescence and DLS analysis. All experiments were performed three times with 5-6 technical replicates each, and the data expressed as mean ± SEM were normalized to percentage fluorescence intensity. Graphs were generated using ORIGIN 2024 software, as previously described [71].

### 4.4. Kinetic Fitting of ThT Fluorescence Curves:

Normalized ThT Fluorescence Traces Were Fitted with a Logistic IV Function Using OriginPro 2024. The curve fittings were restricted to the rising phase (from baseline to approximately 95% of the plateau) to minimize the baseline noise and late-phase drift. The equation used for fitting is:

Y = Y_0_ + (A − Y_0_)/(1 + e^(−k(x − t_50_))),

where Y_0_ is the baseline fluorescence, A is the maximum fluorescence (plateau), k is the apparent rate constant, and t_50_ is the half-time of aggregation. Fitting was performed using the Levenberg–Marquardt algorithm and the resulting parameters (t_50_ and k) were used to compare aggregation kinetics across different experimental conditions. The quality of each fit was verified by evaluating the residuals and the correlation coefficients (R^2^ = 0.92–0.99). For IC_50_ analysis, the % inhibition was calculated using the ThT fluorescence intensity measured at the 48 h endpoint. Tau-seed-induced Aβ1-42 aggregation in the absence of PLGA was considered as the control. The % inhibition at each PLGA concentration was then calculated as:

% inhibition = [(Fcontrol − FPLGA)/Fcontrol] × 100,

where Fcontrol represents the 48 h endpoint ThT fluorescence of tau-seed-induced Aβ1-42 aggregation without PLGA and FPLGA represents the corresponding fluorescence in the presence of PLGA. The % inhibition values obtained with 5, 10 and 20 µM PLGA were plotted against PLGA concentration and fitted using a variable-slope Hill equation:

% inhibition = Emax × [PLGA]^n^/(IC_50_^n^ + [PLGA]^n^),

where Emax represents the fitted maximal inhibitory effect, IC_50_ represents the concentration of PLGA producing 50% of the fitted maximal inhibitory effect, and “n” represents the Hill coefficient. Nonlinear regression analysis yielded the apparent IC_50_ and Emax values for PLGA-mediated inhibition of tau-seed-induced Aβ1-42 aggregation [36]. Each experiment was repeated three times, and the mean values are presented.

### 4.5. Fluorescence Microscopy

A 10 μL aliquot of Aβ1-42 and Aβ42-1 samples was taken from different experimental conditions (i.e., with or without 0N4R/2N4R tau seeds/monomers and PLGA), air-dried on glass slides and stained with ThT solution. The ThT-stained aggregates were visualized using a Nikon Eclipse 90i fluorescence microscope at 20× magnification [36].

### 4.6. Dynamic Light Scattering (DLS)

The PDI of native PLGA nanoparticles determined from DLS analysis was found to be 0.065. The size distribution of various aggregated Aβ samples with or without tau seeds + PLGA seeds was determined after 48 h of incubation using a Malvern Zetasizer-Nano Instrument as described previously [34,35]. A He–Ne laser (λ = 632 nm) detected backscattered light at 173°. The aggregated Aβ samples (with or without 5–20 μM tau seeds + 5–20 μM PLGA) were incubated at 37 °C under constant shaking. Data were collected from 10 consecutive 10 s runs, using 100 μL samples in a 10 mm quartz cuvette. Measurements were made without agitation, and the particle size was calculated using the Stokes–Einstein equation via DTS v6.20 software. The refractive index was assumed equal to those of water (1.33).

### 4.7. Transmission Electron Microscopy (TEM)

The morphological alterations of Aβ samples from various experimental paradigms were evaluated using a Talos F200C TEM. For high-resolution structural analysis, images were acquired at a magnification of 45,000× in Selected Area (SA) mode. Initially, 5 μL of Aβ samples (±tau seeds with or without PLGA) was adsorbed onto glow-discharged, 400-mesh carbon-coated copper grids for 60 s, washed with double-distilled water, and stained with 2% filtered uranyl acetate. The grids were then dried and imaged at 200 kV accelerating voltage using a bottom-mounted Ceta 16M camera between 22 K and 45 K magnification with around −3 μm defocus [35].

### 4.8. Detection of Soluble Aβ Using NanoDrop

To validate the tau-seeded ThT kinetic assay, we quantified soluble Aβ in the absence and presence of tau seeds and native PLGA nanoparticles. Briefly, 10 µM Aβ1-42 was incubated for 48 h with or without 10 µM 0N4R tau seeds in the presence or absence of 20 µM PLGA. In parallel, 10 µM tau seeds and 20 µM PLGA were incubated alone as controls. After incubation, all samples were centrifuged at 14,000 rpm for 30 min and the corresponding supernatants were collected for soluble Aβ quantification. These conditions were used to sediment larger insoluble aggregates; thus, it is possible that some smaller fibrillar/oligomeric species remained in the supernatant. As for the positive control (100% soluble Aβ), freshly prepared 10 µM Aβ1-42 was measured immediately after solubilization, first in DMSO and then in PBS buffer. The absorbance was measured at 280 nm using a NanoDrop spectrophotometer (NanoDrop Technologies model ND-1000, Wilmington, Delaware, USA), and the background absorbance of the buffer was subtracted from all experimental conditions. The results were plotted using Origin 2024 software and obtained from three independent experiments.

### 4.9. 8-Anilino-1-Naphthalene Sulfonate (ANS) Binding Assay

The ANS binding assay was used to monitor hydrophobic surface exposure during protein aggregation, as described earlier [71]. In brief, 50 µM ANS was added to 20 µM PLGA alone or to tau-seed-induced Aβ1-42 aggregates with or without 20 µM PLGA and then incubated for 30 min in the dark to minimize photobleaching. Fluorescence emission spectra were recorded from 400 to 700 nm using a SpectraMax M5 multi-plate reader (Molecular Devices, San Joe, California, USA) with excitation set at 380 nm. For data analysis, ANS emission spectra in the buffer alone were subtracted from each corresponding sample spectrum. This procedure was repeated 3–4 times and the mean values were plotted using the Origin 2024 software.

### 4.10. Fourier Transform Infrared (FTIR) Spectroscopy

An FTIR spectroscopy study was performed to identify the secondary structural vibration of 10 µM 0N4R tau-seed-induced Aβ1-42 (10 µM) aggregation in the absence or presence of 20 µM native PLGA. FTIR spectra were acquired in transmission mode on a Nicolet 8700 FTIR spectrometer (Thermo Fisher Scientific, Waltham, MA, USA) using OMNIC 9.16 software. Spectra were collected over 4000–450 cm^−1^ at 4 cm^−1^ resolution with 32 scans using a DTGS detector and an XT-KBr beamsplitter. For sample preparation, a small volume of each sample was deposited onto an FTIR-transparent silica wafer and allowed to air-dry prior to measurement. Blank water was used as the background. For the analysis of PLGA-containing samples, the absorbance spectrum of native PLGA alone was subtracted prior to normalization. The resulting spectra were then used for comparison of the amide-I region.

### 4.11. Dot-Blot

To validate the effects of PLGA on tau (monomer vs. seeds)-induced Aβ1-42 aggregation, we performed dot-blot assay using Aβ fibrillar-specific OC antibody and total tau-specific Tau5 antibody as previously described [71]. In brief, 10 µM 0N4R tau seeds and monomeric tau (prepared in the absence of heparin) were incubated with or without 10 µM Aβ1-42 in the presence or absence of 20 µM native PLGA for 48 h at 37 °C. Subsequently, treated and untreated tau samples (20 μL) were loaded onto a 0.45 μm nitrocellulose membrane and subjected to vacuum filtration using a 96-well Bio-Dot Microfiltration system. The membranes were stained with Ponceau S to assess sample loading and imaged using a scanner. Subsequently, the membranes were washed with 1X TBST (0.2% Tween 20), blocked with 5% BSA for 2 h at room temperature, and incubated overnight at 4 °C with either OC antibody (1:4000) or total tau Tau5 antibody (1:500) which recognizes the fibrillar conformation of Aβ and specific epitope (i.e., amino acids 218–225) of tau protein, respectively. The membranes were then washed 1X TBST and incubated with appropriate HRP-conjugated secondary antibodies (1:2000) for 2 h at room temperature. Protein signals were developed with an ECL kit and captured using FluorChem E imaging system (Santa Clara, CA, USA). Dot-blot signal intensities were quantified using Image J software 1.54r, normalized to corresponding Ponceau S staining, and then statistically analyzed.

### 4.12. Data Analysis

All kinetic data, collected from a minimum of 3-4 separate experiments each performed with 5-6 replicates, are expressed as mean ± SEM. The data were analyzed using one-way ANOVA followed by Tukey’s post hoc analysis for paired comparisons with a significance level set at *p* < 0.05. *p* values indicate the following significances: * *p* < 0.05; ** *p* < 0.01; and *** *p* < 0.001. All statistical analyses were performed using ORIGIN software.

## Data Availability

The datasets used and/or analyzed during the current study are available from the corresponding author upon reasonable request.

## Supplementary Materials

The following supporting information can be downloaded at: https://www.mdpi.com/article/10.3390/ijms27188152/s1.

## Abbreviations

The following abbreviations are used in this manuscript:

Aβ	amyloid β
AD	Alzheimer’s disease
ANS	8-Anilino-1-Naphthalene Sulfonate binding
APP	amyloid precursor protein
BCA	bicinchoninic acid
DLS	dynamic light scattering
DMSO	dimethyl sulfoxide
DTT	dithiothreitol
HFIP	hexafluoro-2-propanol
MTBD	microtubule-binding domain
NFTs	neurofibrillary tangles
PBS	phosphate-buffered saline
PHFs	paired helical filaments
PLGA	poly(D,L-lactide-co-glycolide)
TEM	transmission electron microscopy
ThT	thioflavin-T
